# Measuring Ventilatory Activity with Structured Light Plethysmography (SLP) Reduces Instrumental Observer Effect and Preserves Tidal Breathing Variability in Healthy and COPD

**DOI:** 10.3389/fphys.2017.00316

**Published:** 2017-05-18

**Authors:** Marie-Cécile Niérat, Bruno-Pierre Dubé, Claudia Llontop, Agnès Bellocq, Lila Layachi Ben Mohamed, Isabelle Rivals, Christian Straus, Thomas Similowski, Pierantonio Laveneziana

**Affiliations:** ^1^Sorbonne Universités, UPMC Université Paris 06, Institut National de la Santé et de la Recherche Médicale, UMRS_1158 Neurophysiologie Respiratoire Expérimentale et CliniqueParis, France; ^2^Assistance Publique-Hôpitaux de Paris, Groupe Hospitalier Pitié-Salpêtrière Charles Foix, Service de Pneumologie et Réanimation Médicale (Département “R3S”, Pôle PRAGUES)Paris, France; ^3^Département de Médecine, Service de Pneumologie, Hôpital Hôtel-Dieu du Centre Hospitalier de l'Université de MontréalMontréal, QC, Canada; ^4^Assistance Publique-Hôpitaux de Paris, Groupe Hospitalier Pitié-Salpêtrière Charles Foix, Service des Explorations Fonctionnelles de la Respiration, de l'Exercice et de la Dyspnée (Département “R3S”, Pôle PRAGUES)Paris, France; ^5^Equipe de Statistique Appliquée, ESPCI Paris, PSL Research UniversityParis, France

**Keywords:** breathing variability, breathing pattern, COPD, healthy, instrumental/observer effect, plethysmography

## Abstract

The use of a mouthpiece to measure ventilatory flow with a pneumotachograph (PNT) introduces a major perturbation to breathing (“instrumental/observer effect”) and suffices to modify the respiratory behavior. Structured light plethysmography (SLP) is a non-contact method of assessment of breathing pattern during tidal breathing. Firstly, we validated the SLP measurements by comparing timing components of the ventilatory pattern obtained by SLP vs. PNT under the same condition; secondly, we compared SLP to SLP+PNT measurements of breathing pattern to evaluate the disruption of breathing pattern and breathing variability in healthy and COPD subjects. Measurements were taken during tidal breathing with SLP alone and SLP+PNT recording in 30 COPD and healthy subjects. Measurements included: respiratory frequency (R*f*), inspiratory, expiratory, and total breath time/duration (Ti, Te, and Tt). Passing-Bablok regression analysis was used to evaluate the interchangeability of timing components of the ventilatory pattern (R*f*, Ti, Te, and Tt) between measurements performed under the following experimental conditions: SLP vs. PNT, SLP+PNT vs. SLP, and SLP+PNT vs. PNT. The variability of different ventilatory variables was assessed through their coefficients of variation (CVs). In healthy: according to Passing-Bablok regression, Rf, TI, TE and TT were interchangeable between measurements obtained under the three experimental conditions (SLP vs. PNT, SLP+PNT vs. SLP, and SLP+PNT vs. PNT). All the CVs describing “traditional” ventilatory variables (R*f*, Ti, Te, Ti/Te, and Ti/Tt) were significantly smaller in SLP+PNT condition. This was not the case for more “specific” SLP-derived variables. In COPD: according to Passing-Bablok regression, Rf, TI, TE, and TT were interchangeable between measurements obtained under SLP vs. PNT and SLP+PNT vs. PNT, whereas only Rf, TE, and TT were interchangeable between measurements obtained under SLP+PNT vs. SLP. However, most discrete variables were significantly different between the SLP and SLP+PNT conditions and CVs were significantly lower when COPD patients were assessed in the SLP+PNT condition. Measuring ventilatory activity with SLP preserves resting tidal breathing variability, reduces instrumental observer effect and avoids any disruptions in breathing pattern induced by the use of PNT-mouthpiece-nose-clip combination.

## Introduction

The measurement of spontaneous breathing provides important information relating to respiratory mechanics (Laveneziana et al., 2011, 2014) and breathing control (Barcroft and Margaria, 1931; Clark and Von Euler, 1972; Milic-Emili and Grunstein, 1976; Lind, 1984). In addition, it allows the quantification of breathing variability and complexity, which are associated with relevant clinical outcomes (Engoren, 1998; Wysocki et al., 2006; Teulier et al., 2013; Dames et al., 2014). However, spontaneous breathing measurements are influenced by the high sensitivity of the human respiratory system to the “observer effect”: measuring breathing suffices to modify it (Gilbert et al., 1972; Askanazi et al., 1980; Weissman et al., 1984; Perez and Tobin, 1985; Western and Patrick, 1988; Han et al., 1997; Fiamma et al., 2007a; Rameckers et al., 2007).

As such, an observer effect-free access to respiratory variables is not straightforward. This is a clinically relevant issue as the mathematical biomarkers derived from spontaneous breathing analyses could open novel perspectives for the evaluation of COPD respiratory mechanics, thoraco-abdominal coupling and severity and therapeutic interventions (Teulier et al., 2013; Dames et al., 2014), as has already been demonstrated in studies on patients with chronic obstructive pulmonary diseases (COPD), in which the study of the intrinsic variability of breathing variables was shown to be related to disease severity (Dames et al., 2014). Accordingly, there is a need for a reliable, contactless method of evaluating breathing variables. Structured light plethysmography (SLP) can be seen as such a method: it allows the measurement of ventilatory activity through the stereoscopic analysis of respiratory-related distortions of a black and white checkered pattern projected on the chest wall and abdomen (Elshafie et al., 2016). We hypothesized that SLP would give access to measurements of breathing pattern closer to ecological conditions than those derived from measurements obtained with a mouthpiece, a PNT and a nose-clip, and we tested this hypothesis by comparing SLP measurements of resting tidal breathing pattern and resting tidal breathing variability performed with and without the PNT-mouthpiece-nose-clip combination in 30 healthy subjects and in 30 COPD patients.

## Materials and methods

### Subjects and patients

Thirty clinically stable COPD (Celli et al., 2004) patients referred for routine pulmonary function testing (PFTs) participated in the study (FEV_1_/VC ratio <5th percentile of the predicted value; Pellegrino et al., 2005), a body mass index (BMI) <30 kg/m^2^, absence of restrictive ventilatory defect (plethysmographic Total Lung Capacity <5th percentile of the predicted value; Pellegrino et al., 2005). Thirty healthy subjects with no history of respiratory or neuromuscular disease also participated in the study.

Anthropometric characteristics and baseline PFTs for COPD patients are summarized in Table 1. The research was carried out in accordance with the principles outlined in the Declaration of Helsinki. The subjects gave their written informed consent and the study received the ethical and legal approval of the appropriate external body (Comité de Protection des Personnes Paris Ile de France VI, CPP/11-10-ID RCB: 2009-A01269-48).

**Table 1:** **Baseline characteristics**.

	**COPD patients**	**Healthy subjects**
*N*	30	30
Age, years	65 (8)	33 (9)
Male gender, n	25	19
Height, cm	169 (10)	175 (9.5)
Weight, kg	70 (11)	69 (13)
BMI, kg/m^2^	25 (3)	23 (3)
GOLD grade, n, age		
1	*n* = 6 age = 67 (7)	
2	*n* = 15 age = 63 (8)	
3	*n* = 6 age = 68 (9)	
4	*n* = 3 age = 63 (3)	
FEV_1_/FVC pre BD	48 (13)	
FEV_1_/FVC post BD	48 (14)	
Post-BD FEV_1_, l	1.7 (0.8)	
Post-BD FEV_1_, %pred	61 (21)	
RV, l	3.6 (1.1)	
RV, % pred	157 (53)	
FRC, l	4.5 (1.4)	
FRC, % pred	137 (36)	
TLC, l	7.2 (1.6)	
TLC, % pred	119 (19)	
DLCO, % pred	57 (22)	

*Data are expressed as mean (± SD). BMI, body mass index; GOLD, Global initiative for Obstructive Lung Disease; BD, bronchodilator; DLCO, diffusion capacity of the lung for carbon monoxide; FEV_1_, Forced expiratory volume in 1 s (Liter); FRC, functional residual capacity; FVC, forced vital capacity; RV, residual volume; TLC, total lung capacity; % pred, percent of predicted value*.

### Study design

**Figure 1:**
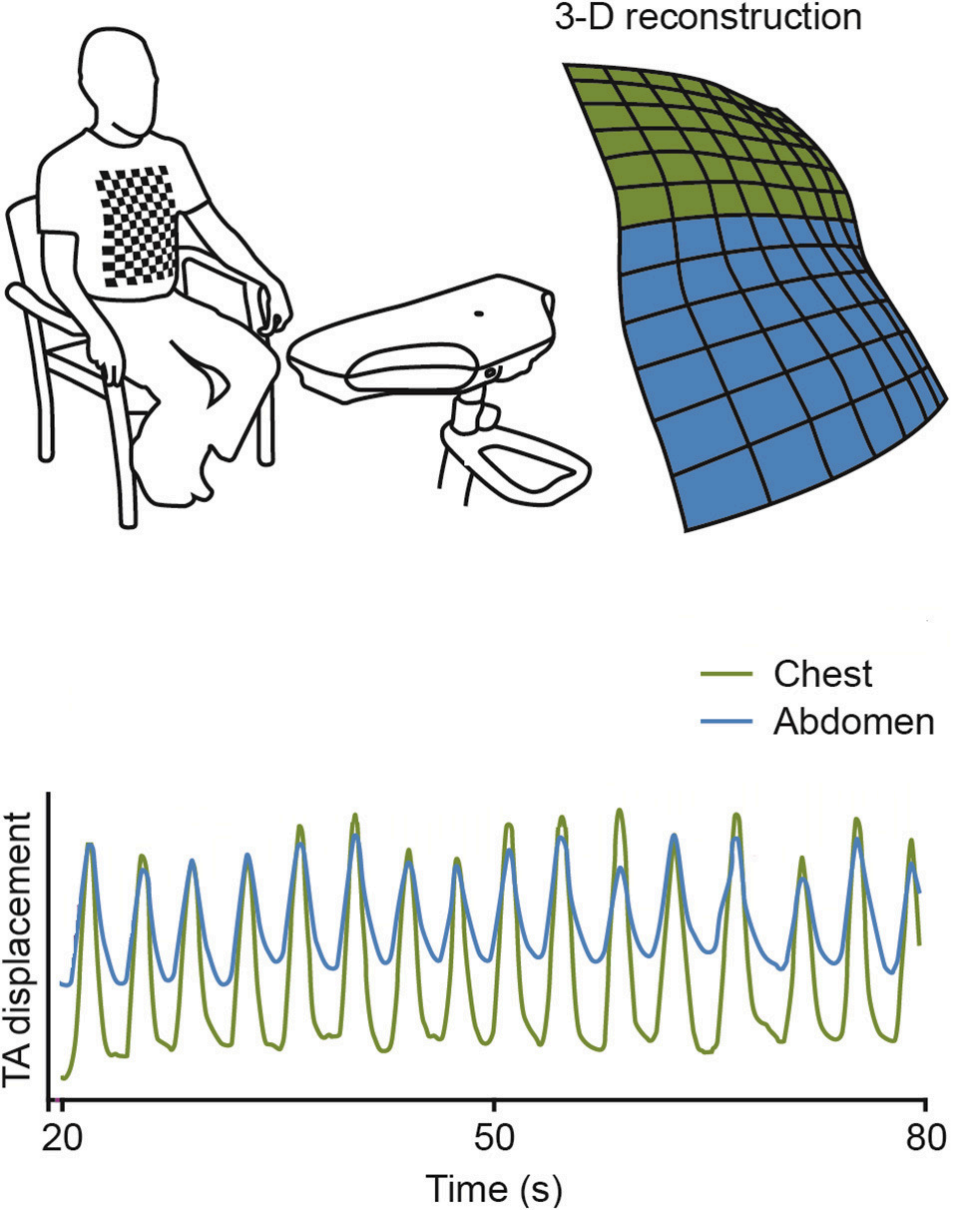
**Schematic illustration of Structured Light Plethysmography (SLP) use in practice**. TA, thoraco-abdominal contribution.

COPD patients carried out only one visit during which they firstly performed standard Pulmonary Function Tests (PFTs) according to recommended standards (MacIntyre et al., 2005; Miller et al., 2005; Wanger et al., 2005); PFT measurements were expressed in absolute value and as percentages of predicted values (Pellegrino et al., 2005). Healthy subjects did not go through lung function testing: the healthy volunteers who participated in the study were subject to strict medical history-taking and clinical examination, the results of which made significant lung function defect most unlikely. Secondly, measurements of chest and abdominal wall movements during quiet tidal breathing were performed using SLP (Thora-3Di®, PneumaCare, Cambridge, UK; Elshafie et al., 2016; Motamedi-Fakhr et al., 2017) in both COPD and healthy subjects. Briefly, subjects were seated on a chair with a headrest and armrests. A structured light grid pattern was projected onto the chest and abdominal wall, and two overhead digital stereoscopic cameras recorded the distortions of this pattern induced by breathing. This information was used to reconstruct breathing pattern from a dedicated algorithm (de Boer et al., 2010). In our study, participants were asked to change into a close fitting white t-shirt that followed the contours of the body or, if they preferred, SLP data acquisition could be performed on the bare chest. Participants were asked to sit upright in a high-backed chair with their neck in a neutral position and their back as straight as possible. They were also asked not to move. The projector was lined up to project the grid of light over the participant's chest and upper abdomen (Figure 1).

Data was collected during 5 min of tidal breathing while subjects were, in random order, (1) breathing normally (SLP condition), and (2) breathing normally through a mouthpiece and low-resistance PNT (M.E.C. PFT Systems Pocket-Spiro, Medical Electronic Construction, Brussels, Belgium) with a nose-clip on (SLP+PNT condition); at this time, data for analysis was generated simultaneously from SLP and from PNT (respectively SLP+PNT condition and PNT condition). The choice of 5 min recording was dictated by technical reasons, such as: (1) the difficulty of having the subjects not to move for 5 min while sitting on a chair with their neck in a neutral position and their back as straight as possible and (2) the need of avoiding artifacts related to saliva due to mouthpiece.

Measurements obtained from PNT and SLP included: respiratory frequency (R*f*), inspiratory and expiratory time (Ti and Te) and total breath time/duration (Tt); specific measurements obtained exclusively from SLP included: time to peak tidal expiratory flow over expiratory time (TPTEF/Te, a measure of airway obstruction and flow-limitation during tidal breathing; Morris et al., 1990; Van Der Ent et al., 1996; Bates et al., 2000), time to peak tidal inspiratory flow over inspiratory time (TPTIF/Ti, a measure of inspiratory flow reserve during tidal breathing; Bates et al., 2000) and thoracic contribution to tidal breathing (T_cont_,%). The investigator(s) responsible of performing SLP and SLP+PNT recordings was/were not involved in the analysis of the results. SLP and SLP+PNT data were anonymized for analysis purpose and interpreted/analyzed in a blinded fashion.

### Statistical analysis

For each data set, the normality of the distribution was checked by using Shapiro-Wilk's test. Values are shown as mean ± standard deviation (*SD*) or median and interquartile range [Q1–Q3] depending on the distribution of data and on the statistical test used.

A Passing-Bablok regression analysis (Passing and Bablok, 1983) was applied to determine whether timing components of the ventilatory pattern (R*f*, Ti, Te, and Tt) were interchangeable between the following experimental conditions: SLP vs. PNT, SLP+PNT vs. SLP, and SLP+PNT vs. PNT. The Passing-Bablok regression is a non-parametric rank regression, where the slope of the regression line is calculated as a shifted median of all possible slopes between pairs of points. It is resistant to outliers, but it gives an unbiased slope estimate only if both methods have constant coefficients of variation (Passing and Bablok, 1983). To evaluate the disruption of breathing pattern and breathing variability in healthy and COPD subjects, comparisons were made only between SLP and SLP+PNT conditions by using Wilcoxon's signed rank test. In all cases, *p* < 0.05 was considered statistically significant. The within subject variability of the different ventilatory variables was assessed through their coefficients of variation (CV). Statistical analyses were performed using Medcalc (MedCalc Software, Ostend, Belgium) and SigmaPlot (Systat Software, San Jose, CA).

## Results

Baseline characteristics of the patients and healthy volunteers are shown in Table 1.

In healthy: according to Passing-Bablok regression, Rf, TI, TE, and TT were interchangeable between measurements obtained under the three experimental conditions (SLP vs. PNT, SLP+PNT vs. SLP, and SLP+PNT vs. PNT). In COPD patients: according to Passing-Bablok regression, Rf, TI, TE, and TT were interchangeable between measurements obtained under SLP vs. PNT and SLP+PNT vs. PNT. Whereas only Rf, TE, and TT were interchangeable between measurements obtained under SLP+PNT vs. SLP.

Table 2 and e-Figures 1, 2 (please refer to the on-line supplement) summarize the Passing–Bablok regression analysis' results along with intercept A values, slope B values and their relative confidence intervals of R*f*, Ti, Te, and Tt and of respiratory mechanical variables obtained exclusively from SLP (T_cont_,%, TPTIF/TI, and TPTEF/TE) between measurements obtained under the three experimental conditions (SLP vs. PNT, SLP+PNT vs. SLP, and SLP+PNT vs. PNT) in healthy subjects and COPD patients.

**Table 2:** **Passing–Bablok regression analysis comparing, in Healthy Subjects and in COPD patients, timing components of the ventilatory pattern (R*f*, Ti, Te, and Tt) and respiratory mechanical variables obtained exclusively from SLP (T_cont_,%, TPTIF/Ti, and TPTEF/Te) between measurements performed under the following experimental conditions: SLP vs. PNT, and SLP+PNT vs. SLP, and SLP+PNT vs. PNT**.

			**Ti (s)**	**Te (s)**	**Tt (s)**	**R*f* (breath/min)**	**T_cont_,%**	**TPTIF/Ti**	**TPTEF/Te**
Healthy Subjects	SLP vs. PNT	Intercept A	−0.47	0.64	0.19	0.75			
		95% CI	[−1.9–0.3]	[−0.3 to 1.3]	[−1.5 to 1.2]	[−3.8 to 5.0]			
		Slope B	1.32	0.70	0.95	0.95			
		95% CI	[0.9 to 2.2]	[0.4 to 1.1]	[0.7 to 1.3]	[0.7 to 1.2]			
		*p* value	*p* = 0.86	*p* = 0.53	*p* = 1.00	*p* = 1.00			
	SLP+PNT vs. SLP	Intercept A	0.32	−0.20	0.13	−0.03	2.16	−0.07	−0.01
		95% CI	[−0.1 to 0.8]	[−1.4 to 0.8]	[−1.1 to 1.1]	[−4.5 to 3.6]	[−2.7 to 7.1]	[−0.3 to 0.1]	[−0.2 to 0.1]
		Slope B	0.83	1.07	0.98	0.99	0.96	1.14	1.17
		95% CI	[0.6 to 1.1]	[0.7 to 1.5]	[0.8 to 1.3]	[0.7 to 1.3]	[0.8 to 1.1]	[0.9 to 1.6]	[0.9 to 1.6]
		*p* value	*p* = 0.86	*p* = 0.86	*p* = 0.86	*p* = 0.16	*p* = 0.67	*p* = 0.67	*p* = 0.93
	SLP+PNT vs. PNT	Intercept A	0.13	0.23	0.12	0.66			
		95% CI	[−0.2 to 0.4]	[−0.1 to 0.6]	[−0.2 to 0.5]	[−0.3 to 1.9]			
		Slope B	1.00	0.86	0.97	0.95			
		95% CI	[0.9 to 1.2]	[0.7 to 1.0]	[0.9 to 1.0]	[0.9 to 1.0]			
		*p* value	*p* = 0.86	*p* = 0.85	*p* = 0.86	*p* = 0.86			
COPD patients	SLP vs. PNT	Intercept A	0.36	0.09	0.49	1.30			
		95% CI	[−0.1 to 0.6]	[−0.4 to 0.5]	[−0.5 to 1.1]	[−3.4 to 4.1]			
		Slope B	0.71	0.97	0.88	0.92			
		95% CI	[0.5 to 1.0]	[0.8 to 1.2]	[0.7 to 1.1]	[0.8 to 1.2]			
		*p* value	*p* = 0.91	*p* = 0.91	*p* = 0.16	*p* = 0.63			
	SLP+PNT vs. SLP	Intercept A	−**0.34**	−0.23	−0.82	−2.34	4.19	−**0.19**	−0.04
		95% CI	**[**−**0.9 to 0.1]**	[−0.9 to 0.3]	[−2.0 to 0.3]	[−6.9 to 0.5]	[−4.2 to 14.5]	**[**−**0.6 to** −**0.0]**	[−0.2 to 0.0]
		Slope B	**1.33**	1.15	1.29	1.10	0.95	1.33	**1.40**
		95% CI	**[1.1 to 1.7]**	[0.9 to 1.5]	[1.0 to 1.6]	[0.9 to 1.4]	[0.8 to 1.1]	[1.0 to 2.1]	**[1.1 to 1.9]**
		*p* value	*p* = 0.91	*p* = 0.63	*p* = 0.91	*p* = 0.91	*p* = 0.63	*p* = 0.58	*p* = 1.00
	SLP+PNT vs. PNT	Intercept A	0.02	−0.09	−0.17	−0.57			
		95% CI	[−0.3 to 0.2]	[−0.4 to 0.1]	[−0.4 to 0.0]	[−1.6 to 0.1]			
		Slope B	1.00	1.06	1.07	1.01			
		95% CI	[0.9 to 1.2]	[1.0 to 1.2]	[1.0 to 1.1]	[0.1 to 1.1]			
		*p* value	*p* = 0.91	*p* = 0.63	*P* = 0.63	*p* = 0.91			

*SLP, Structured Light Plethysmography; PNT, pneumothacograph; Rf, respiratory frequency; TI, Inspiratory time; TE, Expiratory time; TT, total breath time/duration; TPTEF, Time to Peak Tidal Expiratory Flow; TPTIF, Time to Peak Tidal Inspiratory Flow; Tcont,%, Thoracic contribution to breathing; CI, confidence interval. Values shown in bold are not statistically interchangeable according to Passing-Bablok analysis*.

As for comparison between SLP and SLP+PNT conditions, the following variables obtained exclusively from SLP were statistically interchangeable according to the Passing-Bablok regression:

1. Healthy subjects: T_cont_,%, TPTIF/TI, and TPTEF/TE.
2. COPD: T_cont_,%.

As for comparison between SLP and SLP+PNT conditions, the following variables obtained exclusively from SLP were statistically not-interchangeable according to the Passing-Bablok regression:

1. Healthy subjects: none.
2. COPD: TI, TPTIF/TI, and TPTEF/TE.

Table 3 summarizes the differences between variables measured during SLP+PNT condition and during SLP condition in healthy subjects. In most cases, there was no statistically significant difference between the two conditions. In contrast, all the coefficients of variation describing “traditional” respiratory variables (R*f*, Ti, Te, Tt, Ti/Te, and Ti/Tt) were significantly smaller in the SLP+PNT condition. This was not the case for more “specific” SLP-derived variables.

**Table 3:** **Comparison of ventilatory variables between healthy subjects with and without PNT**.

**Healthy**	**SLP**	**SLP+PNT**		***p*-value**
**MEAN**				
R*f*, breath/min	13.5 [11.5–16.2]	13.8 [12.0–15.3]		0.967
Ti, s	1.8 [1.5–2.4]	1.9 [1.8–2.2]		0.77
TE, s	2.6 [2.1–3.0]	2.4 [2.1–2.9]		0.41
TT	4.4 [3.8–5.4]	4.4 [4.0–5.1]		0.73
Ti/TE	0.8 [0.68–0.84]	0.8 [0.74–0.87]		0.18
Ti/TT	0.43 [0.40–0.45]	0.44 [0.42–0.46]		0.08
TPTIF/Ti	0.54 [0.48–0.58]	0.54 [0.44–0.58]		0.57
TPTEF/TE	0.29 [0.23–0.36]	0.32 [0.27–0.39]		**0.016**
T_cont_,%	43.4 [35–48.8]	42.4 [36.9–49.1]		0.61
	**SLP**	**SLP+PNT**	**DELTA (%)**	***p*****-value**
**COEFFICIENT OF VARIATION ANALYSIS**				
R*f*	0.10 [0.08–0.13]	0.07 [0.05–0.09]	−32.5	**<0.001**
Ti	0.11 [0.09–0.18]	0.08 [0.07–0.11]	−20.8	**<0.001**
TE	0.13 [0.09–0.16]	0.08 [0.07–0.11]	−35.7	**<0.001**
TT	0.10 [0.07–0.13]	0.07 [0.05–0.09]	−30.8	**<0.001**
Ti/TE	0.16 [0.12–0.22]	0.11 [0.08–0.15]	−30.2	**<0.001**
Ti/TT	0.09 [0.07–0.11]	0.06 [0.05–0.08]	−29.7	**<0.001**
TPTIF/Ti	0.23 [0.2–0.28]	0.23 [0.20–0.29]	1.1	0.97
TPTEF/TE	0.38 [0.31–0.41]	0.33 [0.28–0.39]	−11.5	0.15
T_cont_,%	0.08 [0.06–0.12]	0.09 [0.06–0.13]	7.9	0.95

*Data are presented as median and interquartile range [Q1–Q3]. Comparisons between SLP and SLP+PNT were made using Wilcoxon's signed rank test. SLP, Structured Light Plethysmography; PNT, pneumothacograph; Rf, respiratory frequency; Ti, Inspiratory time; Te, Expiratory time; Tt, Total breath duration; TPTEF, Time to Peak Tidal Expiratory Flow; TPTIF, Time to Peak Tidal Inspiratory Flow; T_cont, %_, Thoracic contribution to breathing; DELTA, difference between SLP+PNT and SLP measures. Values shown in bold are not statistically interchangeable according to Passing-Bablok analysis*.

Table 4 describes the comparison between measurements performed with SLP+PNT and with SLP only in COPD patients. Most discrete variables were significantly different between the SLP and SLP+PNT conditions and coefficients of variations were significantly lower but one (TPTIF/Ti, *p* = 0.074) when COPD patients were assessed in the SLP+PNT condition.

**Table 4:** **Comparison of ventilatory variables between COPD patients with and without PNT**.

**COPD**	**SLP**	**SLP+PNT**		***p*-value**
**MEAN**				
R*f*, breath/min	15.6 [14.3–21.3]	15.7 [12.3–20.0]		**0.04**
Ti, s	1.4 [1.2–1.8]	1.6 [1.3–1.9]		**0.001**
TE, s	2.2 [1.6–2.6]	2.2 [1.7–2.8]		0.3
TT, s	3.9 [2.8–4.4]	3.8 [3.0–4.9]		**0.048**
Ti/TE	0.77 [0.6–0.8]	0.81 [0.6–0.9]		**0.006**
Ti/TT	0.43 [0.4–0.5]	0.44 [0.4–0.5]		**0.005**
TPTIF/ Ti	0.54 [0.5–0.58]	0.52 [0.5–0.6]		**0.037**
TPTEF/TE	0.24 [0.2–0.3]	0.28 [0.2–0.3]		**<0.001**
T_cont_,%	45.5 [36.3–52.4]	46.1 [37.4–55.2]		0.074
	**SLP**	**SLP+PNT**	**DELTA (%)**	***p*****-value**
**COEFFICIENT OF VARIATION ANALYSIS**				
R*f*	0.12 [0.09–0.16]	0.07 [0.06–0.09]	−37.8	**<0.001**
TI	0.13 [0.1–0.17]	0.09 [0.07–0.11]	−28.6	**<0.001**
TE	0.14 [0.12–0.18]	0.10 [0.08–0.12]	−31.7	**<0.001**
TT	0.12 [0.09–0.13]	0.07 [0.06–0.09]	−35.2	**<0.001**
Ti/TE	0.15 [0.14–0.21]	0.11 [0.09–0.13]	−27.3	**<0.001**
Ti/TT	0.09 [0.08–0.12]	0.06 [0.05–0.07]	−31.3	**<0.001**
TPTIF/Ti	0.22 [0.17–0.27]	0.23 [0.20–0.29]	3.3	0.074
TPTEF/TE	0.40 [0.30–0.45]	0.30 [0.27–0.37]	−25.5	**0.014**
T_cont_,%	0.13 [0.07–0.15]	0.09 [0.05–0.11]	−30.5	**0.008**

*Data are presented as median and interquartile range [Q1–Q3]. Comparisons between SLP and SLP+PNT were made using Wilcoxon's signed rank test. SLP, Structured Light Plethysmography; PNT, pneumothacograph; Rf, respiratory frequency; Ti, Inspiratory time; Te, Expiratory time; Tt, Total breath time/duration; TPTEF, Time to Peak Tidal Expiratory Flow; TPTIF, Time to Peak Tidal Inspiratory Flow; T_con, %t_, Thoracic contribution to breathing; DELTA, difference between SLP+PNT and SLP measures. Values shown in bold are not statistically interchangeable according to Passing-Bablok analysis*.

## Discussion

The novel findings of this study are as follows: (1) while the timing components of the breathing pattern were interchangeable between measurements obtained under the three experimental conditions (SLP vs. PNT, SLP+PNT vs. SLP, and SLP+PNT vs. PNT) in healthy and in COPD, this was not the case in COPD patients for “specific” SLP-derived variables such as TPTIF/Ti and TPTEF/Te; (2) assessing tidal breathing variability at rest with a mouthpiece-noseclip-PNT combination provides results that are significantly different from those derived from SLP alone, namely a non-contact approach.

In the normal subjects that we studied, discrete descriptors of tidal breathing were generally not different between the three methods, but the variability of these descriptors was significantly lower (~20–35% lower) with the mouthpiece-noseclip-PNT combination compared with SLP alone. In the COPD patients, the differences were more marked, with significant differences regarding the discrete variables in addition to their reduced variability, which was significantly lower (~25–38% lower) with the mouthpiece-noseclip-PNT combination compared with SLP alone. This suggests that the variability of breathing descriptors is sensitive to the instrumental component of the observer effect (assuming that in this study the emotional component of the observer effect should have been similar with the two methods, the participants being always aware that their breathing was studied). In other words, our results suggest that measuring ventilatory activity with SLP preserves breathing variability.

### Effects on within subject variability

The within-subject variability of tidal volume and other descriptors of tidal breathing under stable prevailing conditions is a natural property of the human respiratory system that has been described with the very first measurements of respiration by Jules Marey in the nineteenth-century (Marey, 1864; Michaelis, 1966; De Neve, 1983). This variability is interpreted as an indicator of neuromechanical coupling (the tighter the coupling, the lower the variability. Many studies have shown that increased mechanical loads decrease breathing variability, both in an experimental context (Brack et al., 1997, 1998, 2002) and in a clinical context (Wysocki et al., 2006). Breathing variability is also influenced, under constant mechanical conditions, by the intensity of the neural drive to breathe (the higher the drive, the lower the variability). For example, stimulating breathing with carbon dioxide in normal humans considerably decreases the variability of tidal breathing (Fiamma et al., 2007b). In the present study, within subject variability was lower when measures were performed with the mouthpiece-noseclip-PNT combination than with SLP (Tables 3, 4). It seems reasonable to assume that variability was decreased in the PNT condition, and “normal” or “natural” in the SLP condition. Our data cannot disentangle the contribution of altered neuromechanical coupling or increased neural drive to breathe in the observed decrease in variability: nonetheless, altered neuromechanical coupling is unlikely to have occurred in the healthy subjects as confirmed by the absence of change in variability of “specific” SLP-derived variables such as T_cont_, TPTIF/Ti, and TPTEF/Te that relate more to intrinsic respiratory mechanics. Rather, it is possible that the use of a PNT/noseclip was associated with a change in neural to breathe, possibly related to the change in dead space ventilation or airway resistance caused by the device. In COPD patients, clues to an increased drive to breathe and significant change (decrease of 25–30%) in variability of “specific” SLP-derived variables such as T_cont_ and TPTEF/Te that relate more to intrinsic respiratory mechanics were present (Table 4). Few other studies have investigated the impact of the “observer effect” on breathing variability, but our data are in line with previously reported ones with impedance plethysmography (Fiamma et al., 2007a; Rameckers et al., 2007).

### Effects on respiratory mechanics and thoraco-abdominal coupling

Our data demonstrate that the use of PNT-mouthpiece-nose clip combination modifies respiratory mechanics and thoraco-abdominal coupling at rest in COPD patients to a greater extent than in healthy subjects (Tables 3, 4). Of interest, the observed modifications are different in nature and profile, some of them being only captured by SLP. In the healthy subjects, TPTEF/TE was the only variable significantly different between the two conditions (slower expiration in the presence of the PNT). This expiratory index should have been determined by the sole elastic recoil of the lungs (no neural drive, and therefore no possibility for adjustment): finding it altered by an expiratory resistance, even small, is not surprising. In contrast, many variables significantly differed between conditions in the COPD patients. These included timing components of the ventilatory cycle and TPTIF/Ti, TPTEF/TE, and Tcont,% (Table 4). This suggests that the presence of the PNT sufficed to modify the equilibrium previously established in response to the COPD related abnormalities (strong instrumental observer effect). In the literature, the impact of a measurement apparatus involving breathing via the mouth through a PNT has long been identified and evaluated. Mead et al. were the first to address this issue by using body surface derived measurement of breathing activity—contact magnetometers (Mead et al., 1967). With the same approach, Gilbert et al. showed that breathing through a mouthpiece-noseclip-PNT apparatus decreased breathing frequency and increased tidal volume (Gilbert et al., 1972). Similar data have been obtained with different approaches such as head canopy systems (Askanazi et al., 1980; Weissman et al., 1984) and electrical impedance devices (Perez and Tobin, 1985; Western and Patrick, 1988; Han et al., 1997; Rameckers et al., 2007). Perez et al. demonstrated that the observed changes mostly related to the change in breathing route induced by the mouthpiece-noseclip-PNT apparatus (Perez and Tobin, 1985). All these studies were conducted in healthy individuals. The present one is seemingly the first to address this issue in patients in general, and in COPD patients in particular. Our observations invalidate the hypothesis that the COPD-related underlying abnormalities (increased respiratory mechanical impedance and increased drive to breathe at rest) would offset the effects of the measuring apparatus, as in the case of normal subjects during hypercapnia (Fiamma et al., 2007b), altitude-related hypoxia (Rameckers et al., 2007) or exercise (Wagner and Clark, 2016). In the contrary, they show that the changes induced by the measuring device (in respiratory mechanics and/or in breathing route) are sufficient to generate changes in the previously established breathing equilibrium.

### Limitations

This study has certain limitations that need to be acknowledged. Firstly, the emotional/cognitive component of the observer effect was not evaluated (see above). Secondly, the SLP device does not give access to actual measures of tidal volume: we could not evaluate the impact of the observer effect on this very important variable. This, however, is not different from other non-PNT methods of respiratory measurement. Of notice, the SLP approach allows quantification of compartmentalized thoraco-abdominal respiratory movements and their relative contribution to breathing, which other approaches do not. Thirdly, we did not measure variables relating to the neural drive to breathe (occlusion pressure, respiratory muscle electromyograms), which prevents us to fully understand the mechanisms underlying the observed differences. Fourth, different set-up conditions between our study and those published in the literature cited above related to dead space of the mask and/or mouthpiece and spirometer internal resistance may explain the small differences observed in our results. Finally, we did not compare the use of SLP with other “non-traditional” methods of breathing measurement (i.e., optoelectronic plethysmography, wearable/jacket devices). Such a comparison did not fall in the scope of our study but could represent a target for future research, as the comparison of the effects of a true contactless technique such as SLP to other methods that also allow the need for a PNT, but remain in physical contact with subjects could provide additional insights on the impact of the various components of the observer effect on breathing measurement.

## Conclusions and perspectives

Resting tidal breathing variability has been proposed as a clinically useful marker of disease severity (Dames et al., 2014), as a prognostic indicator in critical illness (Engoren, 1998; Wysocki et al., 2006) and as a novel approach to describe the effects of treatments on breathing under ecological conditions (Teulier et al., 2013; Dames et al., 2014). This approach could reveal improvements brought by therapy but of a magnitude too small to remain visible when confronted to the forced expiratory maneuvre “tornado.” Our study shows that the instrumental component of the observer effect can influence the quantification of ventilatory variability in health and disease such as COPD, and can suffice to significantly modify breathing pattern in disease. It is therefore an incentive to consider the use of non-contact devices for the study of tidal breathing as a new way of assessing the physiology of breathing in research and clinical practice.

In conclusion, we can definitively say from our observations that, like traditional techniques, SLP is able to detect different breathing patterns in COPD patients compared with subjects with no respiratory disease. Of importance, the SLP approach not only allows investigators to study breathing pattern in general, but it also gives access to a refined compartmentalized analysis of the thoraco-abdominal behavior. To our knowledge, this is not the case of wearable devices (by the way, very few of them, if any, seem to have been the object of rigorous validation in normal subjects or in patients). This provides support for further investigation into the potential uses of SLP in assessing clinical conditions and interventions, as it has recently been shown in children in whom SLP, a non-contact and non-invasive method for measuring tidal breathing, can differentiate between those with and without airway obstruction and may identify responses to bronchodilator (Hmeidi et al., 2017). Of course further research to confirm these observations is underway, but these preliminary observations are promising.

## Author contributions

PL and TS are the guarantors of the paper, taking responsibility for the integrity of the work as a whole, from inception to published article. In addition PL, TS, MN, and BD contributed substantially to the study design, data analysis and interpretation, and the writing of the manuscript. CL, AB, LL, IR, and CS contributed substantially to the data analysis and interpretation, and the writing of the manuscript.

## Authorship

All the authors meet the International Committee of Medical Journal Editors (ICMJE) recommendations requirements for authorship.

### Conflict of interest statement

TS received an unrestricted grant from PneumaCare Limited to study structured light plethysmography in health and disease. The other authors declare that the research was conducted in the absence of any commercial or financial relationships that could be construed as a potential conflict of interest.
