# Supplementary material for: Measuring Ventilatory Activity with Structured Light Plethysmography (SLP) Reduces Instrumental Observer Effect and Preserves Tidal Breathing Variability in Healthy and COPD
Source: Front Physiol. 2017 May 18;8:316. doi: 10.3389/fphys.2017.00316 (PMC5435806; doi:10.3389/fphys.2017.00316)
Supplement: Supplementary file 1 [file Presentation1.pdf]

ONLINE SUPPLEMENT

Measuring ventilatory activity with structured light plethysmography (SLP) reduces instrumental observer effect and preserves tidal breathing variability in healthy and COPD

Authors:
Marie-Cécile Niérat[1*], Bruno-Pierre Dubé[1,2,3*], Claudia Llontop[1,4], Agnès Bellocq[1,4], Lila Layachi Ben Mohamed[1,2], Isabelle Rivals[1,5], Christian Straus[1,4], Thomas Similowski[1,2$], Pierantonio Laveneziana[1,4$]

Affiliations:
[1]Sorbonne Universités, UPMC Université Paris 06, INSERM, UMRS_1158 Neurophysiologie respiratoire expérimentale et clinique, Paris, France ; [2]Assistance Publique-Hôpitaux de Paris (AP-HP), Groupe Hospitalier Pitié-Salpêtrière Charles Foix, Service de Pneumologie et Réanimation Médicale (Département "R3S", Pôle PRAGUES), Paris, France ; [3]Département de Médecine, service de Pneumologie, Hôpital Hôtel-Dieu du Centre Hospitalier de l'Université de Montréal (CHUM), Montréal, Canada ; [4]Assistance Publique-Hôpitaux de Paris (AP-HP), Groupe Hospitalier Pitié-Salpêtrière Charles Foix, Service des Explorations Fonctionnelles de la Respiration, de l'Exercice et de la Dyspnée (Département "R3S", Pôle PRAGUES), Paris, France ; [5]Equipe de Statistique Appliquée, ESPCI Paris, PSL Research University, Paris, France.

Figure Legends

e-Figure 1 : Passing-Bablok regression scatter diagram with the regression line (solid line), and identity line (x = y, dotted line) for R*f* , TI, TE and TT under the three experimental conditions (SLP versus PNT, SLP+PNT versus SLP and SLP+PNT versus PNT) in Healthy Subjects (e-1A, e-1B, e-1C).

e-Figure 2 : Passing-Bablok regression scatter diagram with the regression line (solid line), and identity line (x = y, dotted line) for R*f* , TI, TE and TT under the three experimental conditions (SLP versus PNT, SLP+PNT versus SLP and SLP+PNT versus PNT) in COPD patients (e-2A, e-2B, e-2C).
