# Supplementary figures and images for: Measuring Ventilatory Activity with Structured Light Plethysmography (SLP) Reduces Instrumental Observer Effect and Preserves Tidal Breathing Variability in Healthy and COPD
Source: Front Physiol. 2017 May 18;8:316. doi: 10.3389/fphys.2017.00316 (PMC5435806; doi:10.3389/fphys.2017.00316)

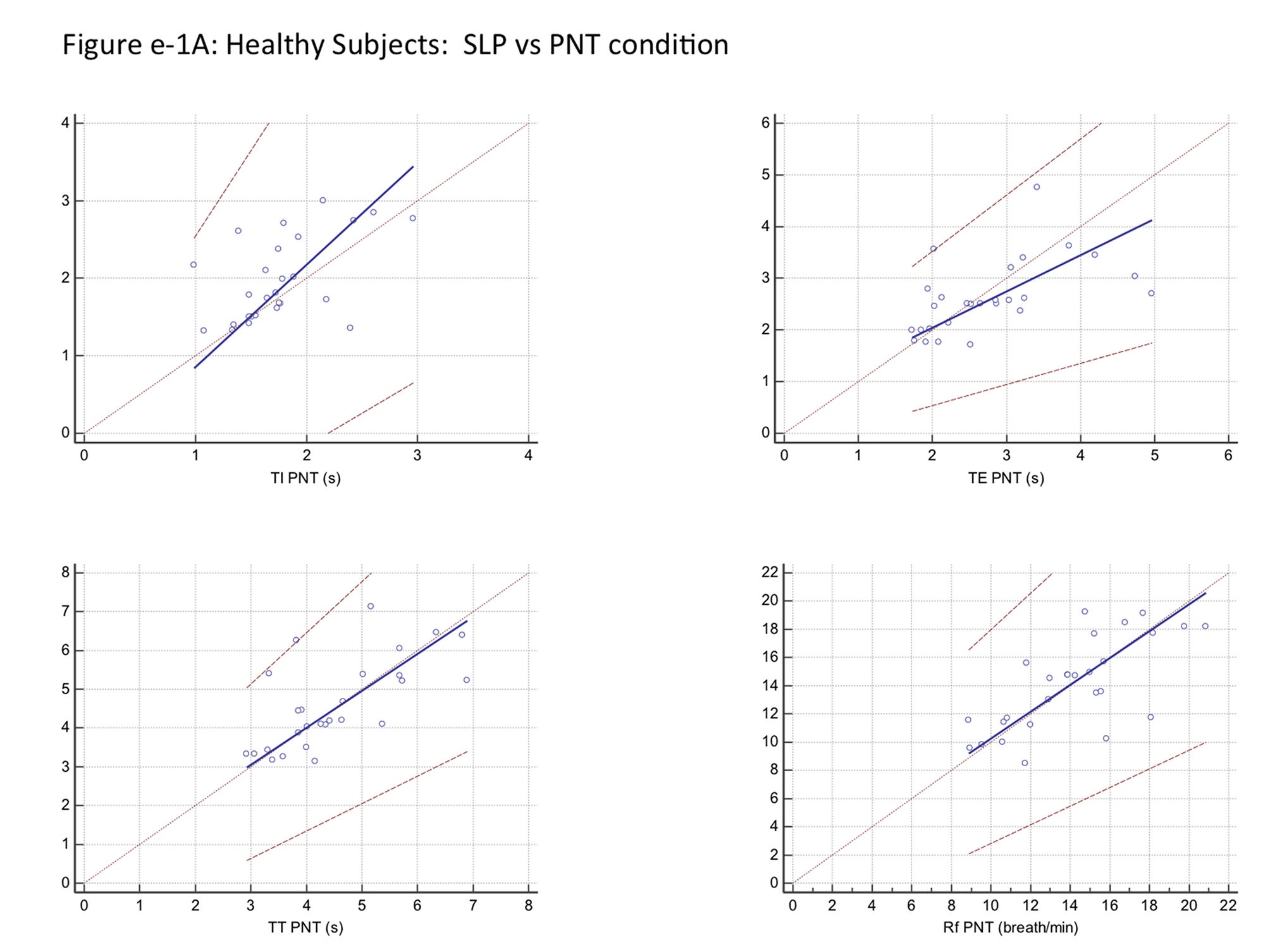

Supplement: Supplementary file 2 [file Image1.TIFF]

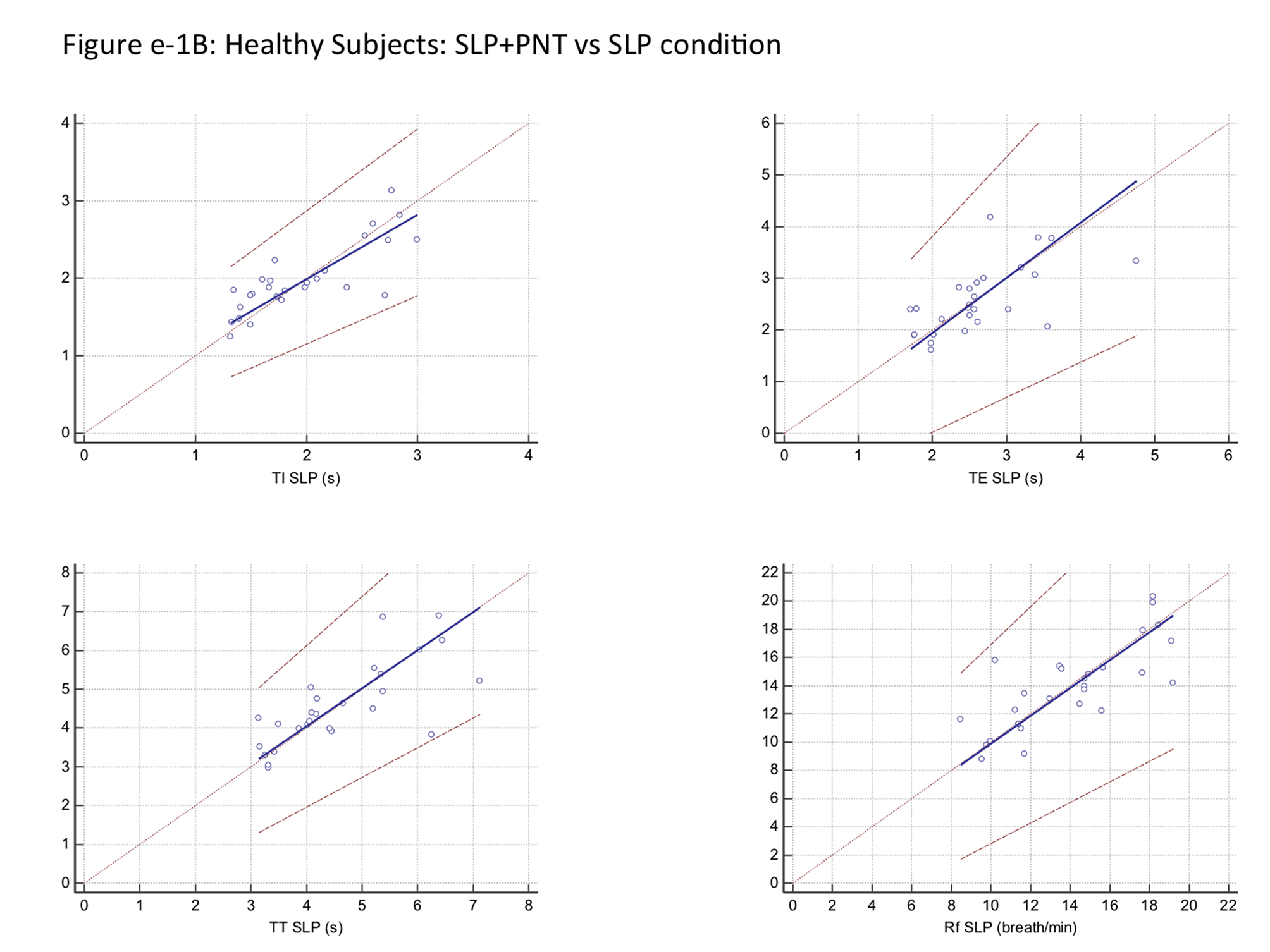

Supplement: Supplementary file 3 [file Image2.TIFF]

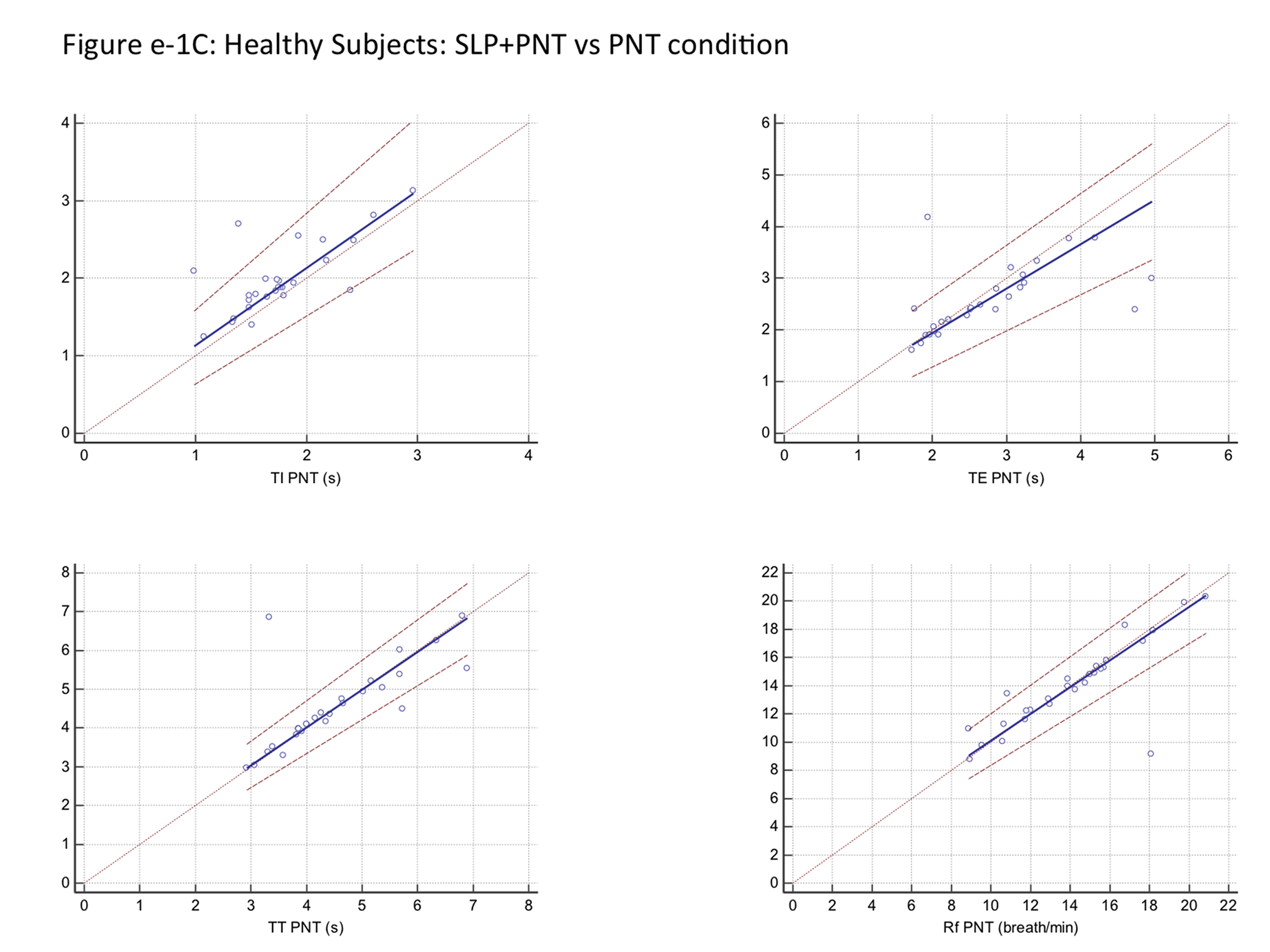

Supplement: Supplementary file 4 [file Image3.TIFF]

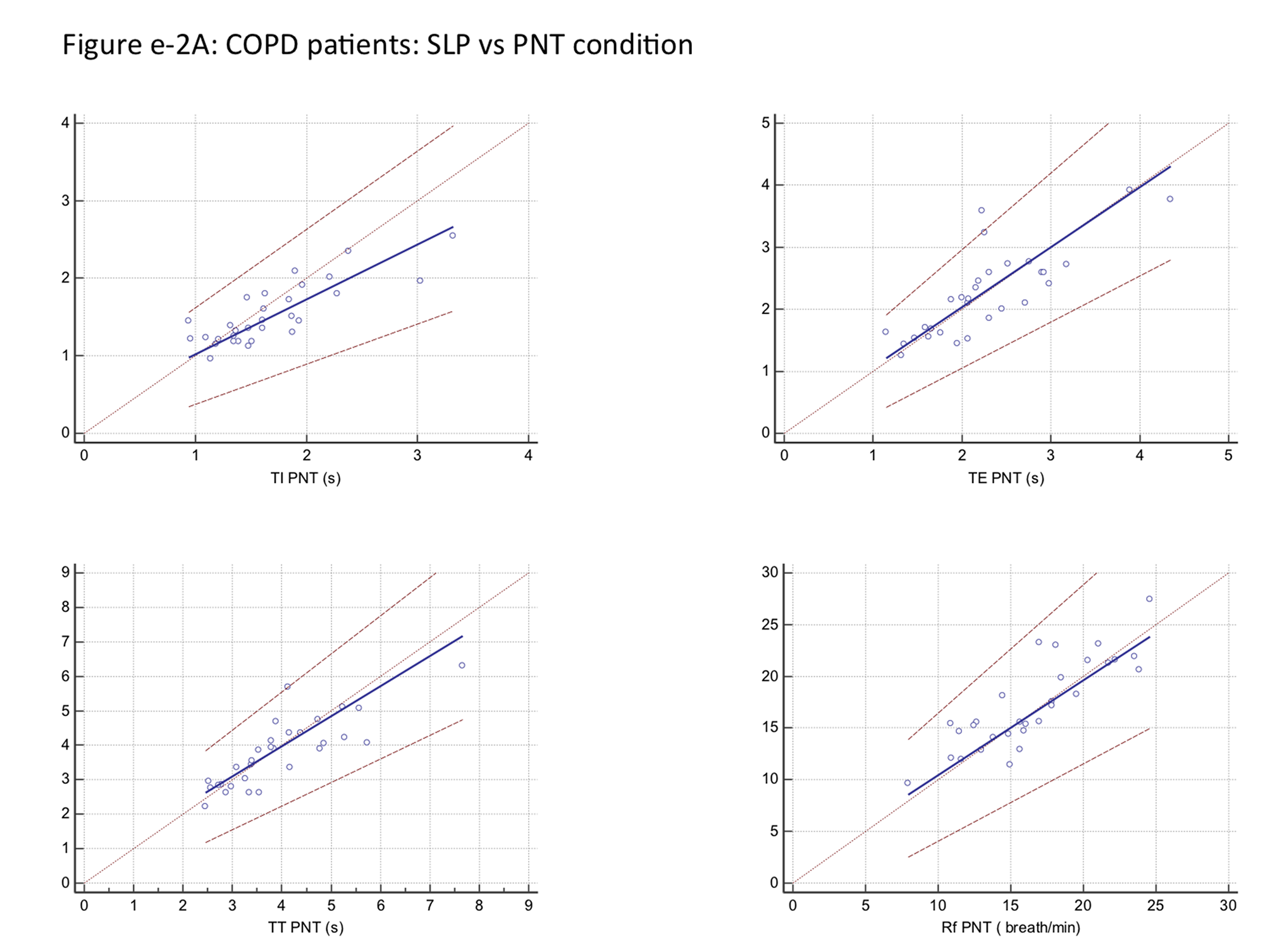

Supplement: Supplementary file 5 [file Image4.TIFF]

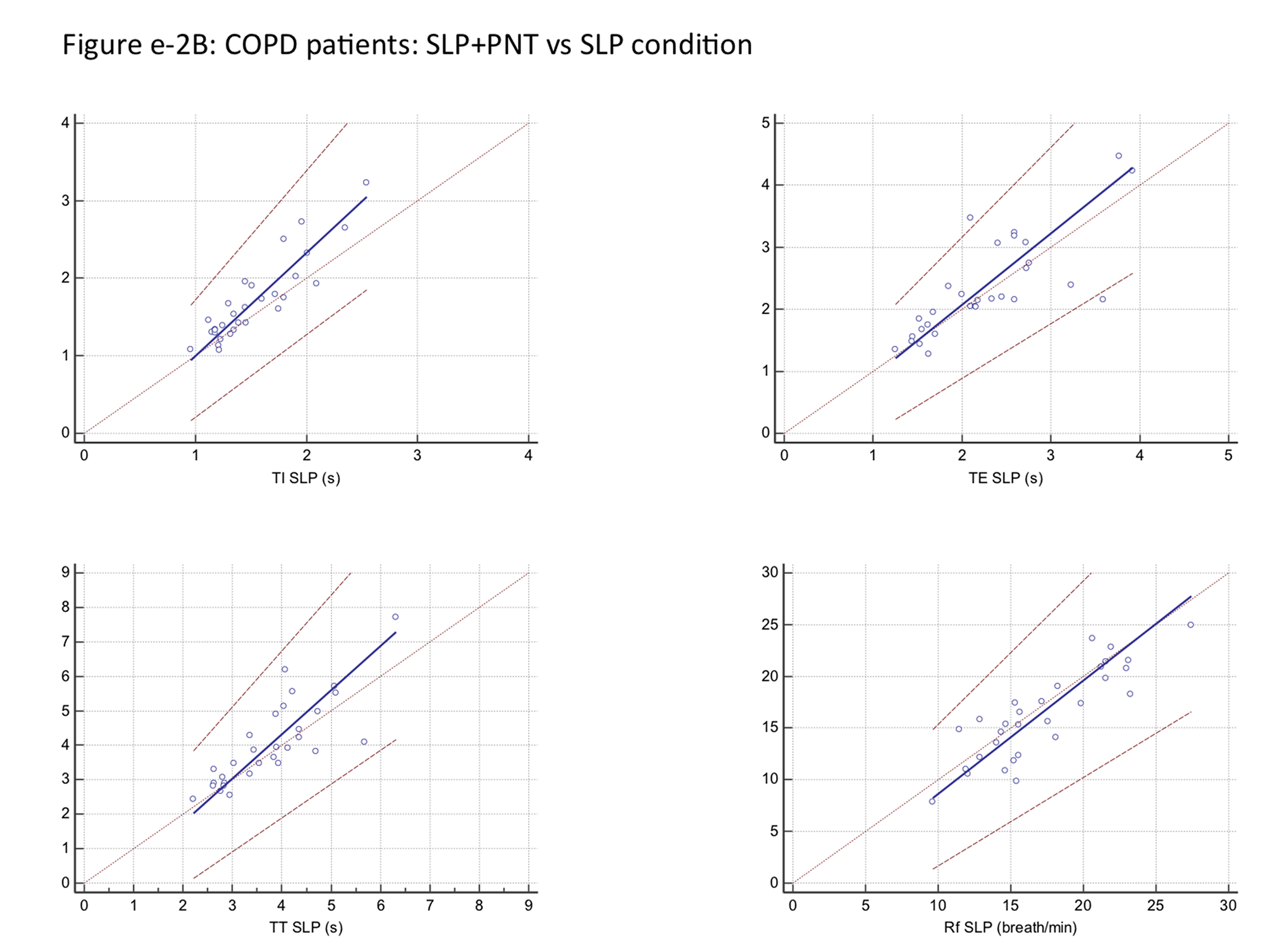

Supplement: Supplementary file 6 [file Image5.TIFF]

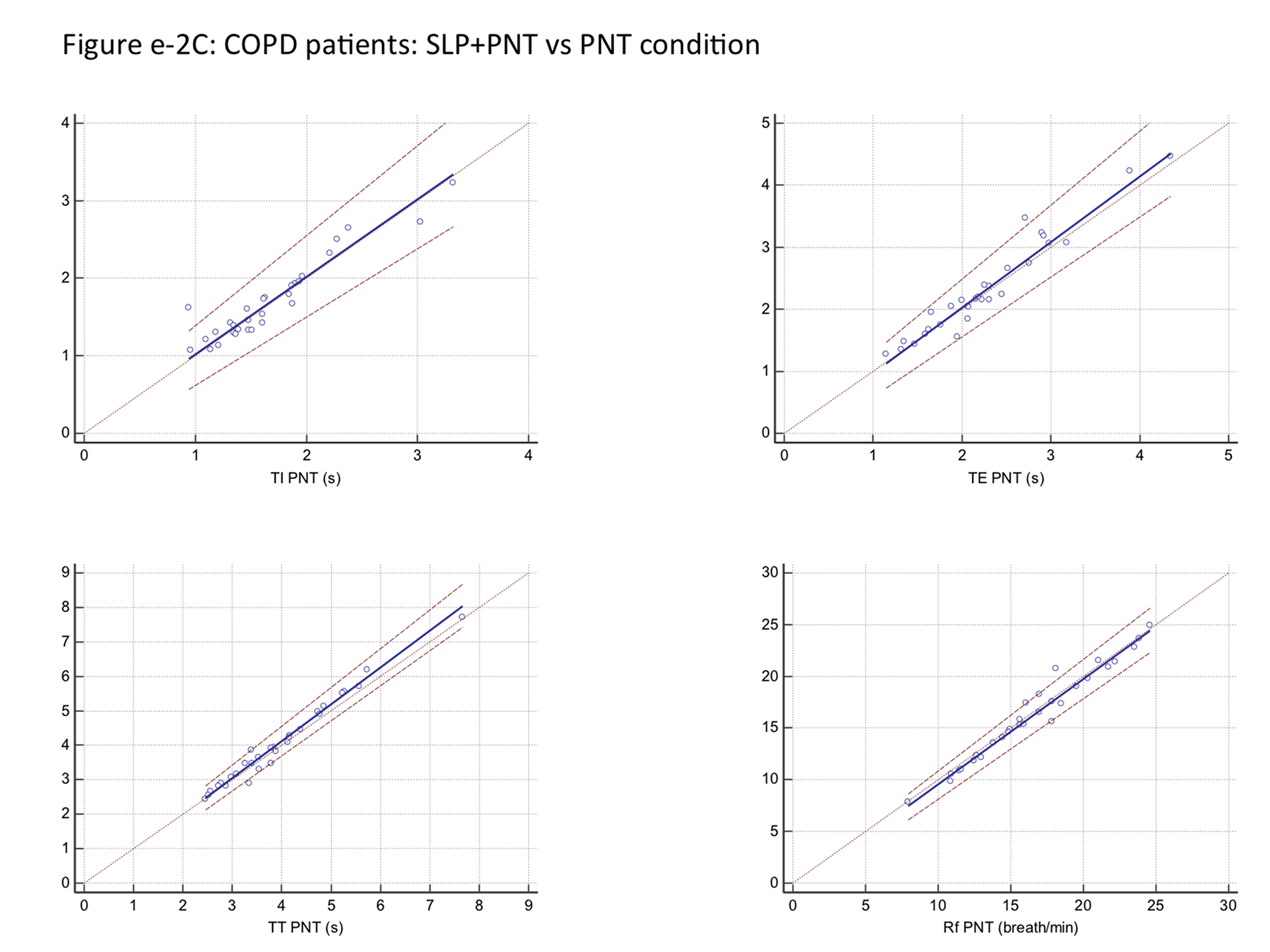

Supplement: Supplementary file 7 [file Image6.TIFF]
